# Modelling *C9orf72*-Related Amyotrophic Lateral Sclerosis in Zebrafish

**DOI:** 10.3390/biomedicines8100440

**Published:** 2020-10-21

**Authors:** Gabrielle Fortier, Zoé Butti, Shunmoogum A. Patten

**Affiliations:** 1 INRS-Centre Armand-Frappier Santé et Biotechnologie, Laval, QC H7V 1B7, Canada; fortier.gabrielle@gmail.com (G.F.); zoe.butti@iaf.inrs.ca (Z.B.); 2 Centre d’Excellence en Recherche sur les Maladies Orphelines—Fondation Courtois (CERMO-FC), Université du Québec à Montréal (UQAM), Montréal, QC H2X 3Y7, Canada

**Keywords:** ALS, zebrafish, *C9orf72*, motoneuron

## Abstract

A hexanucleotide repeat expansion within the *C9orf72* gene is the most common genetic cause of amyotrophic lateral sclerosis (ALS) and its discovery has revolutionized our understanding of this devastating disease. Model systems are a valuable tool for studying ALS pathobiology and potential therapies. The zebrafish (*Danio rerio*) has particularly become a useful model organism to study neurological diseases, including ALS, due to high genetic and physiological homology to mammals, and sensitivity to various genetic and pharmacological manipulations. In this review we summarize the zebrafish models that have been used to study the pathology of *C9orf72*-related ALS. We discuss their value in providing mechanistic insights and their potential use for drug discovery.

## 1. Introduction

Amyotrophic lateral sclerosis (ALS) is a devastating neurodegenerative disease characterized by the degeneration of the upper motor neurons in the motor cortex and the lower motor neurons in the spinal cord. The disease typically begins with a loss of muscle strength and gradually leads to paralysis. Eventually, most patients will die within two to five years after diagnosis due to weakness of the respiratory muscles [1,2]. The disease occurs with an estimated incidence of 1:100,000 and a lifetime risk of 1:1000. To date, there is no existing treatment that provides significant clinical benefits. Only Riluzole and edaravone are currently approved and can prolong patient survival to a very limited degree [3,4]. Most cases of ALS are sporadic (sALS), but about 10% are familial (fALS) and have a strong inherited link [5].

In 1993, *SOD1* (Cu-Zn superoxide dismutase 1) was the first gene to be associated with fALS [6]. Over the past two decades, several mutations in more than two-dozen genes have been discovered to in ALS, including *TARDBP* (transactive response to DNA binding protein 43kDa), *FUS* (fused in sarcoma) [7,8,9] and *C9orf72* (C9orf72-SMCR8 complex subunit) [10,11]. Importantly, an abnormal GGGGCC repeat expansion in the first intron of the *C9orf72* gene is the most common genetic cause in ALS patients [10,11]. About 40% of patients with the familial form of ALS and 8–10% of sALS patients have the *C9orf72* hexanucleotide repeat expansion [12]. The hexanucleotide repeat expansion in the *C9orf72* gene is also associated with frontotemporal dementia (FTD). Some reports have suggested that ALS patients harbour a higher number of repeats than FTD patients [13,14]. Nevertheless, how a repeat expansion in the *C9orf72* gene causes neurodegeneration in both ALS and FTD is still poorly understood. Since the discovery of *C9orf72* mutation, several animal models including the worm (*Caenorhabditis elegans)*, the drosophila, the mouse and the zebrafish have been used to investigate the *C9orf72* form of ALS [15,16,17,18].

With about 70% homology between human and zebrafish genes, this animal model appears to be a powerful organism for studying the *C9orf72* form of ALS in a vertebrate [19]. Zebrafish as a model is easily genetically manipulated, thus ideal for creating stable transgenic lines to investigate loss- or gain-of-function phenotypes [20,21]. Moreover, the translucency of this model allows simple visualization of the organs/systems of the embryo and to some extent at adult stage in zebrafish *casper* mutant. In the last few years, the zebrafish has also proven itself for high-throughput drug screening and confirm the capacity of a molecule to rescue neuronal and motility phenotypes, including in ALS models [22]. The use of this vertebrate model may thus offer an exciting opportunity to identify new therapies for the *C9orf72* form of ALS.

This review summarizes what is currently known about the pathogenesis of ALS associated with the *C9orf72* gene. An overview of the use of zebrafish as a model system is provided and the different zebrafish models that have been developed over past couple years to study the pathological mechanisms of ALS related to the *C9orf72* gene are then discussed.

## 2. The *C9orf72* Gene Associated with ALS

The human *C9orf72* gene consists of 11 exons and, by alternative splicing, encodes 3 variant transcripts, of which the first and third are translated into the same isoform (481 amino acids) and the second variant is translated into a shorter protein (222 amino acids) [10]. Its sequence is highly conserved between different species including common model systems such as mouse (98.1%) and zebrafish (76.0%). An abnormal GGGGCC repeat expansion in the first intron of the *C9orf72* gene is the most common genetic cause in ALS patients [10,11]. The GGGGCC hexanucleotide repeat expansions reside between exon 1a and 1b of the gene. The majority of healthy individuals have ≤11 hexanucleotide repeats in the *C9orf72* gene while hundred to several thousand repeats have been reported in ALS/FTD heterozygous carriers [23,24]. The pathological expansion repeat-length threshold has not been clearly established but an arbitrary cut-off of carrying 30 repeat-alleles is used in most studies. In the last few years, many developments have occurred to explain how the GGGGCC repeat expansion in the *C9orf72* gene is associated with ALS. There are currently 3 proposed pathogenic mechanisms by which the repeat expansions, both sense and antisense (GGGGCC)_n_, cause ALS. These involves two toxic gain-of-function mechanisms, such as RNA foci accumulation [25] and protein toxicity by aberrant dipeptide repeat protein accumulation [26,27]. Alternatively, repeat expansions cause a decrease in *C9orf72* mRNA and protein expression, suggesting loss-of-function by C9orf72 haploinsufficiency may also contribute to *C9orf72* ALS.

Reports have shown that abnormal repeat expansion within the C9orf72 causes haploinsufficiency due to its interference with gene transcription [10,18]. In addition, cytosine hypermethylation of CG dinucleotides in CpG islands (regions of DNA with significantly higher frequency of CG sequence that regulate transcription at their associated promoters) is an important epigenetic modification that could lead to gene silencing [28]. Indeed, several studies have observed decreased levels of *C9orf72* mRNA and protein in *C9orf72* patients CNS tissues, lymphoblastoid cell lines and neurons derived from patient-derived induced pluripotent stem cells (IPSCs) [10,29,30]. To date, the function of the C9orf72 protein still remains unclear, but it has been reported to contain a DENN domain (differentially expressed in normal and neoplasia). The proteins of this family are highly conserved and are GDP/GTP exchange factors (GEF) that activate Rab-GTPases, suggesting that C9orf72 is involved in GTPase activity and the regulation of vesicular transport [31]. Indeed, recent studies have reported that the C9orf72 protein function in a complex with the WDR41 and SMCR proteins as a GEF for Rab8 and Rab39 [32,33]. C9orf72 has also been proposed to play a role in autophagic flux [32,34,35], endosomal trafficking [36,37,38] and regulating AMPA receptor levels [39]. For example, the SQSTM1 protein (p62) is an autophagy protein and knockdown of C9orf72 in human cell lines showed an accumulation of cytoplasmic aggregates of p62, reflecting an impairment in the autophagosome-lysosome system in these cells [36,40]. This observation is consistent with p62-positive inclusion bodies found in ALS and FTD cases [41]. Additionally, knockdown of C9orf72 in human cell lines have shown to result in cytoplasmic aggregates of the RNA binding protein, TDP-43 [36,40]. C9orf72 can interact directly with Importin-β1 or Ran-GTPase [42] and may play a role in nucleocytoplasmic transport [43,44]. Consequently, defects in this pathway may in part account for the mislocalization of TDP-43 from the nucleus to cytoplasm and TDP-43 pathology in ALS.

One of the gain-of-function mechanism hypotheses is that the transcription of the repeat expansion in sense and antisense direction leads to the accumulation of RNA foci [45]. It has been reported in previous studies that patients with the *C9orf72* form of ALS have an accumulation of RNA foci in the brain and the spinal cord [10,25,45,46,47,48,49]. The RNA foci recruit the RNA-binding proteins, inducing their mislocalization and consequently preventing them from performing their normal function, leading to altered RNA metabolism [50]. For example, Lee et al. in 2013 [51] showed that the hnRNP-H protein binds directly to GGGGCC RNA and colocalizes with RNA foci. A list of proteins that bind RNA foci has been identified from previous in vivo and in vitro studies [52].

The other toxic gain-of-function mechanism is the formation of dipeptide repeat proteins (DPRs) due to associated non-ATG (RAN) translation of transcripts in the sense and antisense directions. There are five different DPRs, Gly-Ala (poly-GA), Gly-Arg (poly-GR), Gly-Pro (poly GP), Pro-Ala (poly-PA) and Pro-Arg (poly-PR), forming insoluble aggregates in the nucleus and cytoplasm of the cells [25,26,27]. Dipeptides containing arginine appear to be particularly toxic to cells, they tend to bind the nucleolus and disrupt RNA splicing and ribosomal biosynthesis [53]. For example, research has shown that poly-GR colocalize with ribosomal subunits, thus affecting translation [54]. Poly-PR, on the other side, seem to bind the nuclear pores and block the nuclear transport of RNA and proteins [55].

The relative contribution of these 3 mechanisms remains to be established as well as the molecular pathways leading to motor neuron degeneration. It is however becoming clearer that haploinsufficiency might contribute to the disease process in synergy with the gain-of-toxicity mechanism [56]. Animal models can then provide answers on the different mechanisms involved and consequently help to develop a specific treatment for patients with *C9orf72* ALS.

## 3. Zebrafish as A Model System

Zebrafish as a vertebrate model has received favourable attention from clinicians owing to its many advantages for the study of disease. The rapid, external development of translucent embryos together with low-cost husbandry has sparked the establishment of several models of human diseases in zebrafish [20], and these models often closely resemble the human condition [57]. Additionally, the zebrafish genome has been sequenced [19] and more than 80% of zebrafish genes have a high degree of conserved gene structures across vertebrate species as well as 50–80% amino acid identity with most human homologs, including homologs for over 70% of disease-causing genes. The zebrafish are also especially powerful for genetic manipulation. The majority of their genes can be manipulated by gain- and loss-of-function approaches and consequently makes the zebrafish model system particularly valuable for the study of genetic diseases such as ALS.

The easiest and first approach used to study loss-of-function mutations in zebrafish is the injection of antisense morpholino oligonucleotides (MO) to transiently block translation or splicing [58]. This method only allows the phenotype to be observable over a short period of time and can also leads to off-target toxicity [59,60]. This is why this technique is more useful for drug testing at the embryonic or larval stages [60]. As for the study of gain-of-function mutations, mRNA injection also provides a transient mutation and consequently presents the same limitation as the injection of antisense morpholino oligonucleotides, i.e., the impossibility to study late-onset phenotypes [60]. The Tol2 transposon system is also popular for integrating a DNA sequence into the zebrafish genome, but it is often necessary to out-crossing over several generations to obtain the desired stable transgenic line [61,62,63]. However, the development of stable transgenic models does have some limitations, such as ectopic expression, toxic overexpression and variability due to genetic background [64]. Genomic engineering tools, including site-specific transgenesis such as Cre-loxP and the Gal4/UAS system, are an exciting approach for genetic model of ALS in zebrafish [65,66]. New genome-editing techniques, including transcription activator-like effector nucleases (TALENs) and clustered regularly interspaced short palindromic repeats (CRISPR), have been developed in the last few years and significantly facilitate the study of loss- or gain-of-function mutations in zebrafish [67,68]. It is now possible to rapidly generate a zebrafish line with the wanted mutation that will be inherited in order to study its phenotype.

The zebrafish has emerged as a very attractive model for the study of neurological diseases. The basic structure of central nervous system in zebrafish has all the major domains found in the mammalian brain and they produce the same neurotransmitters such as glutamate, GABA, serotonin, dopamine, histamine and acetylcholine [69,70]. Despite some notable differences in the size of the zebrafish brain, the overall cognitive processing and sensory and retinotectal pathways, share an overall homology with humans [71]. Noteworthily, the hippocampus is essential for spatial memory in mammals and is derived developmentally from the medial pallium of the telencephalon [72]. In zebrafish, unlike mammals, the telencephalon is everted and the lateral pallium appears to be structurally homologous to the mammalian hippocampus [73]. Importantly, many of the genes implicated in human neurodegenerative diseases, such as ALS, have been identified in zebrafish. The zebrafish motor system has also some similarities to humans [74,75]; making it relevant model for motor neuron diseases [76,77]. However, an important difference between zebrafish and human is that there is no direct telencephalic projections to the spinal cord (i.e., corticospinal tract) in fish [69]. The zebrafish spinal cord shows a comparable organization to human spinal cord with groups of motor neurons located in specific regions of the spinal cord and innervating specific muscle fibers. Particularly, zebrafish consist of two classes of motoneurons, primary motor neurons and second motor neurons, based on their formation time and target musculature [78]. Primary motor neurons are localized relatively dorsally with large cell bodies and thick axons and they innervate the dorsal, middle, and ventral trunk musculature. On the other hand, secondary motor neurons are located more ventrally in the motor column with smaller cell bodies and thinner axons than primary motor neurons and they innervate the dorsal and ventral musculatures. The zebrafish secondary motor neurons are comparable to human alpha motor neurons and innervate ventral as well as dorsal muscle fibers. Of note, the zebrafish primary motor neurons do not have an equivalent in human and likewise, the human gamma motor neurons are not present in zebrafish. The gamma motor neurons are found in species that have limbs or limb like structures since it is involved in proprioception [69].

Several ALS-causing genes have been studied in zebrafish using loss- or gain-of-function approaches. For instance, overexpression of mutant but not wild-type human *SOD1* in zebrafish leads to short motor axons with premature branching associated with deficient swimming in response to touch [79]. Expression upon mRNA injection of human *TARDBP* mRNA containing one of three missense mutations (*TARDBP*^G348C^, *TARDBP*^A315T^ or *TARDBP*^A382T^), but not wild type *TARDBP* at the same level of expression, resulted in motor behavioural defects and hyperbranched ventral root projections to trunk musculature [80]. Injection of human mutant *FUS* mRNA resulted in disrupted nuclear import [81] and accumulation in cytosolic stress granules [82] and either mRNA or MO injection resulted in locomotor deficits and ventral root projection abnormalities [83]. Since the discovery of *C9orf72* mutation as the most common genetic cause of ALS, it has received much attention and has also evidently been modelled in zebrafish.

Zebrafish has only 1 *C9orf72* orthologue (zgc:100846; *c13h9orf72*) and similarly to human C9orf72 protein-coding transcripts, the zebrafish also possesses 3 protein-coding transcripts giving rise to 2 protein isoforms if different size [18]. Here, we review different *C9orf72* zebrafish models (Table 1) that have provided new insights into the pathogenesis of ALS since the abnormal repeat expansion within the *C9orf72* gene was identified in 2011 [10,11].

## 4. *C9orf72* Zebrafish Models

### 4.1. Loss-of-Function Zebrafish Models

A zebrafish MO-based model of *c9orf72* was the first in vivo model to support the loss-of-function hypothesis [18]. The knockdown model was generated using a MO to block the translation of the protein. MO knockdown of *c9orf72* in zebrafish resulted in motor neuron axonopathy, a disturbed arborization and shortened axons at early developmental stages. It also led to motor deficits, in particular, abnormalities in spontaneous and evoked swimming were observed in the knockdown model. Importantly, these phenotypes could be rescued with the expression of human *C9orf72* mRNA, confirming that they were due to the direct effects of loss-of-function of *c9orf72*. This zebrafish knockdown model exhibited important motor behavioural defects as observed in the disease, allowing us to think that the loss-of-function has a contribution in ALS pathogenesis, but is not exclusive. Of note, no major morphological defects and no experiments examining cytoplasmic aggregation of TDP-43 were reported in this zebrafish knockdown model.

Subsequently, Yeh et al. [87] developed in 2018 two constructs in which two portions of the functional domain DENN of *c9orf72* were deleted to study its function and to confirm the specificity of the loss-of-function. One of the constructs consisted of only the upstream part of the domain (c9orf72^u-DENN^) and the other contained the central region (c9orf72^c-DENN^). The models showed an altered neuronal network and inhibition of the response to stimuli. This work demonstrated that the DENN domain is essential to the function of *c9orf72* and that a truncated domain has a dominant negative effect. These models were also able to confirm that a deficiency of *c9orf72* affects axon formation in the hindbrain and spinal motor activity. In addition, an increase in the number of neuronal cells in apoptosis was noted. The models showed significantly reduced GTPase activity compared to controls, which is necessary for vesicular formation and transport. These results suggest the importance of *c9orf72* for crucial neuronal functions and synapse formation. They reported that the knockdown of Tp53 was able to rescue the apoptosis phenotype but did not completely restore the motility deficit. They then concluded that *c9orf72* regulates neuronal apoptosis through Tp53. The study by Yeh et al. [87] also established a deletion construct of Cyclin G1 (an N-terminal deletion) (*ccng1*^Δ1-40^), and when injected into *c9orf72*-deficient embryos, it rescued neuronal apoptosis, axonal deficits and motility. This finding suggests that Cyclin G1 upregulation is involved in the pathogenesis of *c9orf72*-deficient embryos. Thus, the C9orf72-Cyclin G1-Tp53 cascade has an important role in the mechanism behind the disease. Importantly, the zebrafish model with the GTPase activity domain deleted from the C9orf72 protein is sufficient to produce a neurological phenotype similar to the symptoms of ALS patients.

To better understand the role of *C9orf72* loss-of-function in ALS pathogenesis, our group recently generated a stable zebrafish line with a reduced expression of c9orf72 [88] using a microRNA-based gene-silencing approach developed for zebrafish [89]. Unlike MO-based knockdown approach, transgenic zebrafish lines that stably express microRNAs designed to target knockdown desired genes of interest have no apparent non-specific toxic effects [90]. Reduced *c9orf72* function in zebrafish resulted in motor defects, muscle atrophy, motor neuron loss and mortality in early larval and adult stages [88]. TDP-43 form aggregates in neurons, glial cells [91] and axial skeletal muscle [92] and this TDP-43 pathology is a hallmark of ALS. Using a specific antibody that recognizes the highly homologous human TDP-43 ortholog in zebrafish [93], we importantly observed a mislocalization of TDP-43 from the nucleus to the cytoplasm in skeletal muscles of the *c9orf72* loss-of-function model; consistent with TDP-43 pathology in ALS. Analysis of the structure and function of the neuromuscular junctions (NMJs), revealed a significant reduction in the number of presynaptic and postsynaptic structures and an impaired release of quantal synaptic vesicles at the NMJ in the *c9orf72* loss-of-function model. Furthermore, a strong downregulation of the synaptic protein, SV2a, as well as marked reduction in the number and size of Rab3a-positive synaptic puncta at NMJ were observed upon reduced *c9orf72* function in zebrafish, resulting in a reduced rate of synaptic vesicle cycling at the NMJ. Altogether, findings from this zebrafish model suggest that loss-of-function mechanisms may underlie defects in synaptic function at NMJ in ALS.

### 4.2. Gain of Function Zebrafish Models

#### 4.2.1. Hexanucleotide Repeats

Soon after the discovery of *C9orf72* mutation, Lee et al. (2013) [51] used the zebrafish to determine whether expanded GGGGCC transcripts might be toxic and sequester RNA binding proteins in vivo. They particularly injected EGFP constructs containing 8×, 38× or 72× repeats into zebrafish embryos to test the length-dependance GGGGCC toxicity. The construct was then expressed only in small portions of the embryos and in a mosaic distribution. While the injection of 8x GGGGCC DNA vector did not increase the number of cells in apoptosis at 24 h post-fertilization, injection of 38× and 72× GGGGCC DNA vectors in zebrafish led to a significant increase in the number of apoptotic cells. Noteworthy, zebrafish injected with 72× GGGGCC repeats displayed a higher rate of apoptosis compared to those injected with 38× GGGGCC repeats. However, RNA foci were only detected in embryos injected with 72× repeats. These embryos were also caspase-3-positive, with clear nuclear condensation and fragmentation, and supporting cell death due to injection of long repeat expansions. This study was thus able to demonstrate in vivo using the zebrafish model that the expression of longer repeats produces RNA foci that initiate cell apoptosis.

The gain-of-function zebrafish C9orf72 model developed by Swinnen et al. in 2018 [85] complements previous studies by showing that overexpression of 35×, 70× or 90× GGGGCC repeats led to motor axon abnormalities but not overexpression of ten or less repeats. These motor axon defects consisted primarily of reduced axonal growth and aberrant branching at 30 h post-fertilization. This study also reported for the first time that the same deficit was noticed with the injection of 70× hexanucleotide antisense repeat RNAs but injection of 35× GGGGCC repeats was less toxic. Importantly, RNA foci were observed in zebrafish injected with 90× sense and 70× antisense repeats. To further test whether gain-of-function mechanism relies on RNA toxicity or toxic DPR, or both, they also generated 50× repeats of DPRs containing an ATG start codon. Interestingly, zebrafish expressing DPRs containing arginine, specifically GR and PR, exhibited motor axon abnormalities. However, their analyses indicated that repeat RNA toxicity was independent of DPR toxicity. Since RNA foci are known to bind and sequester RNA-binding proteins, Swinnen and colleagues further took advantage of their model to investigate whether different RNA binding proteins such the Pur-alpha protein [48,50] were also able to bind the toxic RNA repeats, as well as the involvement of p62, a protein involved in autophagy [94]. Expression of Pur-alpha inhibited the development of motor axonal defects induced by sense repeat RNA in zebrafish. It also decreased RNA foci formation and increased the levels of p62 protein. The study demonstrated that, through its modulating effect on p62, Pur-alpha prevented axonopathy induced by repeat RNAs in zebrafish. Importantly, the use of zebrafish as model system provided insights in the occurrence of RNA toxicity independently of DPR toxicity in the pathogenesis of the *C9orf72* form of ALS.

A stable C9orf72 transgenic zebrafish model expressing the hexanucleotide repeat expansion was recently developed and characterized by Shaw and colleagues in 2018 [21]. A DNA construct consisting of 89 hexanucleotide repeats under the ubiquitin promotor was injected into the zebrafish embryos to generate the C9orf72 transgenic. Two viable lines were generated (2.2-2 zebrafish line and 2.2-7 zebrafish line) and both exhibited an accumulation of RNA foci and DPRs in muscle and central nervous system (CNS). DPRs were formed by conventional ATG-dependent translation and RAN translation, and in sense and antisense directions. Further characterization of the two 2.2 lines revealed that 2.2-7 zebrafish had a marked reduction in survival rate at 15 days postfertilization and started to exhibit motor behavioural defects as of 5 days postfertilization. Behavioural (swimming) deficits worsened during adulthood and the fish had reduced body weight, consistent with phenotypes observed in ALS. Additionally, muscle atrophy, loss of motor neurons, cognitive impairment, and early mortality in young adults as observed in patients with the *C9orf72* form of the disease were also noted in the zebrafish model. They also reported that the heat shock response (HSR) was activated in the model, as found in ALS patients, and this activation correlated with the disease progression [95]. This stable C9orf72 transgenic zebrafish model presented the most pathological hallmarks of the ALS and it appeared to be a powerful organism for drug screening.

#### 4.2.2. Dipeptide Repeat Proteins (DPRs)

Zebrafish transgenic UAS responder lines with an ATG codon forcing the translation of the poly-GA protein (GA80-GFP) or without an ATG codon (ggggcc80-GFP) were generated by Ohki et al. in 2017 [84] using the Tol2 transposon system. They were interested in establishing a model expressing poly-GA, since this DPR was found in higher quantities in the brain of patients with *C9orf72* repeat expansions [27,96]. First of all, both lines presented a pathological hallmark of *C9ORF72* ALS, consisting of the presence of RNA foci in neurons within the spinal cord. The expression of ggggcc80-GFP had a mild toxicity in zebrafish while the expression of GA80-GFP was highly toxic. Indeed, these fish displayed severe pericardial edema, reduced circulation of red blood cells, and aggregates of GA80-GFP exclusively in the muscles. On the other hand, zebrafish expressing GA80-GFP did not have significant differences in axon length and did not show any vascular patterning defects. However, the endothelial cells in this model were found to be thinner and less well-structured due to a lack of blood perfusion. Ohki and colleagues also injected a MO to specifically targets poly-GA translation, and not the repeat expansion, which this resulted in the rescue of the severe pericardial edema phenotype. This model was used to confirm that poly-GA is highly toxic in zebrafish and suggests the possibility of targeting DPRs as a therapeutic strategy.

After determining that GR was the most toxic of the DPRs, and that 200 GR repeats were largely sufficient to cause significant developmental and motor deficits, Swaminathan et al. [86] developed in 2018 a stable transgenic zebrafish model expressing GR repeats associated with *C9orf72.* They overexpressed 100 GR repeats under the control of the upstream activation sequence (UAS) using the Tol2 transposon system. In the construct, a GFP sequence under the *cmlc2* promoter was used to allow easy identification of the transgenic embryos. Gal4 driver lines under three different promoters were crossed with this transgenic line for cell-specific expression of GR repeats-Hsp: Gal4 for ubiquitous expression after a heat shock, mnx: Gal4 for motor neuron expression, and elavl3: Gal4 for neuronal expression. Ubiquitous expression of GR in zebrafish resulted in severe morphological and motor deficits. On the other hand, GR expression only in motor neurons, led to significant motors deficits with no gross morphological abnormalities. In addition, a decreased in motor neuron length, and an increased in apoptosis cells in the spinal cord were observed in zebrafish expressing GR specifically in motor neurons. However, GR repeat expression did not seem to affect the development of motor neurons. In this study, the zebrafish DPR model demonstrated that GR repeat expression is sufficient to induce motor dysfunction and shortening of motor neuron length in a similar manner to what is observed in ALS patients.

Cytoplasmic accumulation of TDP-43 is a significant neuropathological hallmark in C9-ALS patients. There is indeed a clear association between TDP-43 accumulation and degeneration as well as clinical phenotype [97]. However, none of these gain-of-function zebrafish studies described above reported the presence of cytoplasmic TDP-43 in modelling C9orf72-ALS. Additionally, in these zebrafish models the levels of the C9orf72 protein were not examined. It would have been relevant to check whether expressing the abnormal expansion repeat or dipeptide repeat proteins (DPRs) in zebrafish has an impact on C9orf72 protein levels since some studies have shown a decrease in brain of ALS patients [98].

## 5. Conclusions

ALS is a fatal motor neuron disease and there is an urgent need to develop and assess more effective therapeutics. The GGGGCC hexanucleotide repeat expansion in the *C9orf72* gene is the most common mutation in both familial and sporadic cases of ALS. The generation of *C9orf72* zebrafish models have provided novel insights into the pathogenesis of C9-ALS. It is, however, important to point out that the signature GGGGCC hexanucleotide repeat expansion motif in the *C9orf72* is only present in human and its closest relative, the chimpanzee [99]. Although, the expression of pathogenic GGGGCC repeat expansions in zebrafish result in RNA foci and DPR formation that are toxicity as in ALS, perhaps the best approach to accurately model *C9orf72*-related ALS in zebrafish, is to attempt knock-in the repeats in intron 1 of the zebrafish *c9orf72* with CRISPR genome editing approaches in the future.

In addition to providing a better comprehension of why the hexanucleotide repeat expansion in the *C9orfF72* gene is pathogenic, the zebrafish has the advantage of being a powerful organism for the screening of therapeutic compounds compared to other animal models. The zebrafish C9orf72 model in the study by Shaw et al. in 2018 [21] is a successful example of its use for the discovery of new drugs in a short period of time. They were able to confirm that Ivermectin, a compound that was effective in their SOD1 zebrafish screen, also decreases HSR activation in the C9 zebrafish model, as well as Riluzole [21,100]. Zebrafish are consequently a powerful tool for drug-discovery by allowing rapid drug screening. The prospect of zebrafish for drug discovery opens new avenues using zebrafish ALS models for finding treatments.

## Figures and Tables

**Table 1:** Summary of the different zebrafish C9orf72 models and their distinct phenotypes.

Study	Mechanism	Stable or Transient	Method(s)	Cellular and Behavioral Phenotype
Ciura et al., (2013) [18]	Loss of function	Transient	Morpholino knockdown C9orf72	Axonopathy (disturbed arborization and shortened axons of motor neuron axons) Moto deficits (abnormalities of spontaneous and evoked swimming)
Lee et al., (2013) [51]	Gain of function	Transient	Overexpression (38× and 72× G_4_C_2_)	Increase of apoptotic cells throughout 24 h embryo RNA foci formation with 72× G_4_C_2_
Ohki et al., (2017) [84]	Gain of function	Stable	UAS responder line expressing 80 GGGGCC without an ATG codon (ggggcc80-GFP) was crossed to the Gal4 driver line SAGFF73A for ubiquitous expression	RNA foci formation A mild cardiac phenotype
	Gain of function	Stable	UAS responder line expressing 80 GGGGCC with an ATG codon (GA80-GFP) was crossed to the Gal4 driver line SAGFF73A for ubiquitous expression	RNA foci formation A severe cardiac phenotype A reduced circulation of red blood cells Aggregates of GA80-GFP found in musculature
Swinnen et al., (2018) [85]	Gain of function	Transient	mRNA Overexpression (35×, 70× and 90× G_4_C_2_ and C_2_G_4_)	Axonopathy (disturbed arborization and shortened axons of motor neuron axons) RNA foci formation
Swaminathan et al., (2018) [86]	Gain of function	Stable	UAS transgenic line overexpressing 100 GR repeats was crossed to Gal4 driver lines under three different promoters: Hsp: Gal4 for ubiquitous expression after a heat shock, mnx1: Gal4 for motor neuron expression, and elavl3: Gal4 for neuronal expression.	Reduction in motor neuron length Increase apoptotic cells in the spinal cord No effect on motor neuron development An impaired motor functions Reduction in swimming behaviour Respond poorly to touch
Yeh et al., (2018) [87]	Loss of function	Transient	Overexpression of 2 deletion variants, one containing only de upstream DENN domain and one containing only the central DENN domain (c9orf72^u-DENN^ and c9orf72^c-DENN^)	Altered neuronal network Axon formation in the hindbrain altered Response to stimuli is inhibited Spinal motor activity altered Increase of apoptotic cells Reduced of GTPase activity
Shaw et al., (2018) [21]	Gain of function	Stable	Zebrafish model expresses 89 *C9orf72* hexanucleotide repeat expansions	RNA foci formation in muscles Antisense and sense DPRs in muscles and CNS Altered swimming behaviour Reduced weight gain Mortality Muscle atrophy Motor neuron loss Heat shock response is activated
